# Multi-View Hypergraph Contrastive Learning for Uncertainty-Aware circRNA–Disease Association Prediction

**DOI:** 10.3390/biology15212013

**Published:** 2026-11-05

**Authors:** Yu-Hao Zhou, Xiao-Peng Ji, Zhu-Hong You, Lei Wang, Chang-Qing Yu

**Affiliations:** 1 School of Electronic Information, Xijing University, Xi’an 710123, China; 2 School of Computer Science and Technology, China University of Mining and Technology, Xuzhou 221116, China; 3 Guangxi Key Lab of Human-Machine Interaction and Intelligent Decision, Guangxi Academy of Sciences, Nanning 530007, China; 4 School of Computer Science, Northwestern Polytechnical University, Xi’an 710129, China

**Keywords:** circRNA–disease association, hypergraph convolution, contrastive learning, graph neural network, uncertainty quantification

## Abstract

Circular RNAs (circRNAs) have a covalently closed structure and high stability in complex biological samples, making them promising disease biomarkers. However, the sparsity and imbalance of known circRNA–disease associations (CDAs) necessitate computational approaches for CDA prediction. Competition among multiple circRNAs for shared miRNA pools gives rise to group-level ceRNA regulation, motivating the modeling of higher-order relationships among nodes. Three challenges remain in existing methods: insufficient modeling of higher-order cooperative relationships in similarity networks, incomplete integration of complementary predictive signals, and a lack of sample-level uncertainty awareness in fusion decisions. To address these challenges, we propose MHUCDA, a CDA prediction framework that integrates multi-view hypergraph contrastive learning with uncertainty-aware dual-branch fusion. Hypergraph convolution is used instead of conventional pairwise graph convolution to capture higher-order cooperative regulatory relationships in similarity networks. Cross-view contrastive learning enforces semantic consistency across multiple similarity views. A dual-branch architecture independently models candidate associations from two complementary perspectives, semantic propagation and local structural features, while a gating network dynamically assigns per-sample branch weights. Dual-branch prediction divergence is used as an empirical indicator of relative prediction risk, and predictions for high-divergence samples are adjusted toward a neutral reference value. On the CircR2Disease benchmark under five-fold cross-validation, MHUCDA achieves an AUC of 0.9687 and an AUPR of 0.9623, with consistent performance in separate within-dataset evaluations on three additional datasets. Uncertainty stratification analysis reveals an AUC gap of 0.1263 between the highest- and lowest-confidence groups, supporting the use of branch divergence for empirical risk stratification. Case analysis links selected high-confidence predictions to experimentally verified ceRNA regulatory axes, supporting further investigation of model scores for candidate prioritization.

## Simple Summary

Circular ribonucleic acids are ring-shaped molecules that help regulate how cells work and are associated with various diseases. Finding these associations through laboratory experiments can be costly and time-consuming. We developed a computational method to help identify promising links between these molecules and diseases. The method combines several types of similarity information and examines both broad patterns across the data and local connections around each possible link. It also uses disagreement between two prediction approaches to identify predictions that may be less reliable. Tests on four public datasets showed that the method could effectively rank disease-related links, while predictions with greater agreement generally performed better. Published studies supported 22 of the 25 high-confidence candidate links examined. These findings suggest that the method can help researchers prioritize candidates for further laboratory testing and recognize predictions that require greater caution.

## 1. Introduction

Circular RNAs (circRNAs) are a class of covalently closed RNA molecules generated from precursor mRNAs through back-splicing [1]. This circular topology renders them resistant to exonuclease-mediated degradation, enabling stable persistence and reliable detection in complex biological samples, including serum and exosomes [2]. CircRNAs participate in multiple layers of gene-expression regulation, including microRNA (miRNA) sponging, RNA-binding protein (RBP) interactions, and transcriptional regulation, and have been implicated in the onset and progression of numerous diseases [3]. For example, circMTO1 suppresses hepatocellular carcinoma (HCC) progression by sponging miR-9 and thereby upregulating p21 expression [4]. Similarly, BCRC-3 inhibits bladder cancer cell proliferation through the miR-182-5p/p27 signaling axis. Nevertheless, the curation of validated circRNA–disease associations (CDAs) has lagged behind the rapid discovery of circRNAs, resulting in pronounced data sparsity and class imbalance [5]. Computational CDA prediction is therefore essential for systematically prioritizing candidate associations and guiding experimental validation.

CircRNA-mediated miRNA sponging can involve interactions among multiple regulators. Within competing endogenous RNA (ceRNA) networks, multiple circRNAs often compete for shared miRNA pools, forming group-level cooperative regulatory relationships in which functional outcomes depend on the collective behavior of node sets rather than individual pairwise interactions [6]. This group-level organization is reflected in circRNA similarity networks, where functionally related circRNAs tend to associate with similar disease profiles because they participate in shared regulatory programs. Pairwise graph convolution, which uses node pairs as the basic modeling unit [7], is therefore insufficient for capturing these higher-order cooperative patterns [8]. The two main sources of predictive signals, semantic propagation through shared neighbors and local structural features of candidate pairs, are complementary, and their relative reliability varies across candidate associations according to evidence density [9]. Consequently, fixed-weight fusion is suboptimal; without explicit uncertainty awareness, fusing divergent signals may yield overconfident predictions for associations supported by limited evidence [10].

Recent computational methods for CDA prediction can be broadly grouped into three categories. The first includes association matrix completion and multi-source feature reconstruction approaches, which use known CDA matrices to infer latent association patterns through matrix factorization, feature projection, or similarity network reconstruction, such as NMFMSN [11] and MNDCDA [12]. The second comprises heterogeneous graph representation learning methods, which model multiple biomolecule types within a unified network and use graph attention or multi-type node interaction mechanisms to enhance node representations, such as GEHGAN [13] and MTGCDA [14]. The third focuses on multi-view collaboration and complex relationship modeling, combining multi-view information with higher-order structural representations, as exemplified by RPMVCDA [15]. Although these methods have collectively advanced CDA prediction, they remain limited in three key aspects: the granularity of higher-order relationship modeling, the completeness of multi-view predictive signals, and sample-level awareness in fusion decisions.

Hu et al. used multilayer graph convolution to predict herb–disease associations [16]. MHUCDA instead represents groups of similar nodes through hyperedges and aligns multiple views through contrastive learning. DGHNE enhances a heterogeneous biomedical network and applies propagation to identify candidate disease genes [17]. MHUCDA addresses circRNA–disease associations using learned multi-view hypergraph representations and complementary prediction branches.

Li et al. integrated miRNA, gene and disease subnetworks into a gene-mediated tripartite heterogeneous multiplexing network, using a non-equilibrium random walk to infer miRNA–disease associations [18]. This study illustrates the value of multi-source integration [19], whereas MHUCDA combines hypergraph contrastive embedding with adaptive dual-branch fusion.

AttnGen uses attention-guided masking to learn nucleotide-level saliency for genomic sequence classification [20], providing position-level explanations that differ from the association-level risk ranking considered here. Joghataee et al. applied machine learning to identify rare fungal and tuberculous infections in hospitalized patients, addressing class imbalance and explaining feature contributions [21]. This clinical setting provides context for minority-class prediction and interpretation, whereas MHUCDA prioritizes candidate circRNA–disease associations using prediction scores and branch disagreement.

Three unresolved methodological problems underlie these limitations. First, cooperative regulatory relationships in circRNA similarity networks are inherently group-level behaviors among multiple nodes rather than pairwise interactions [22]. Graph convolution, which uses node pairs as the basic modeling unit, is therefore insufficient for fully capturing such group-level patterns [23]. Second, semantic propagation signals and local structural signals are complementary: the former capture indirect associations through multi-hop meta-paths, whereas the latter extract features from direct neighborhoods [24,25,26]. Because neither signal type can substitute for the other, relying on either alone leads to incomplete association evidence. Third, the reliability of these two signal types varies asymmetrically across candidate associations [27]. Fixed-weight fusion is therefore suboptimal, and when the two signals diverge substantially, fusion mechanisms without uncertainty awareness are prone to overconfident predictions [28]. These considerations motivate an integrated framework that combines representation learning, complementary prediction branches, and uncertainty-aware fusion.

To this end, we propose MHUCDA, a CDA prediction framework based on multi-view hypergraph contrastive learning and uncertainty-aware dual-branch fusion. MHUCDA replaces conventional pairwise graph convolution with hypergraph convolution, thereby modeling cooperative regulatory relationships as group-level propagation processes over hyperedges, and imposes cross-view contrastive constraints to enforce semantic consistency across multiple similarity views. The MHM and HHN branches model candidate associations from two complementary perspectives: semantic propagation and local structure. The divergence between dual-branch predictions is used as an empirical indicator of relative prediction risk. A gating network dynamically assigns branch weights for each sample, and uncertainty-aware calibration adjusts high-divergence predictions toward a neutral reference value.

**Figure 1:**
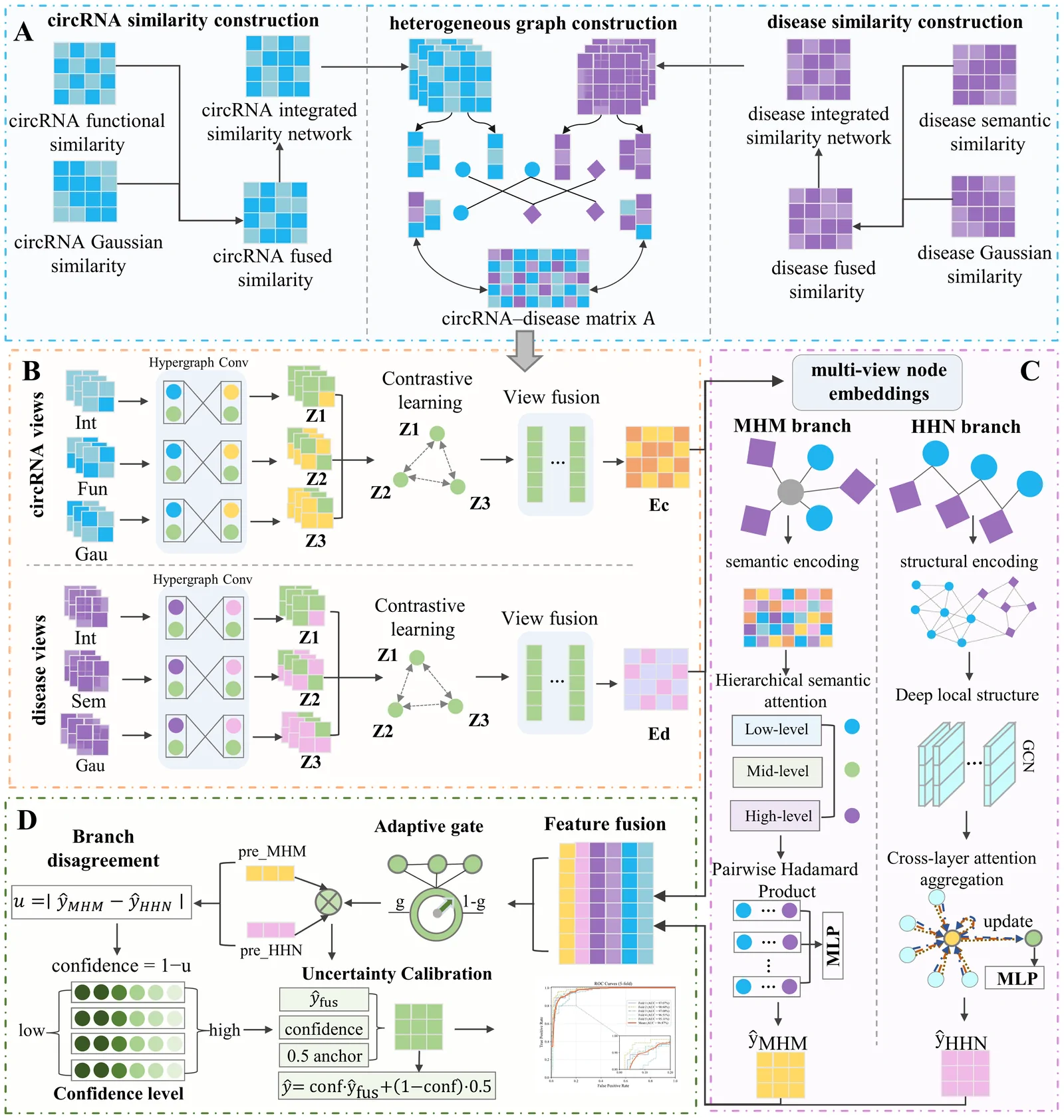
Overall framework of MHUCDA for CDA prediction. (**A**) Multi-source similarity construction and heterogeneous graph assembly: similarity information of circRNAs and diseases is integrated to construct a heterogeneous graph, which serves as the unified model input. (**B**) Multi-view hypergraph contrastive embedding: hypergraph convolution models group propagation processes underlying cooperative regulation. Cross-view contrastive constraints are enforced to ensure semantic consistency across multiple similarity views. (**C**) Dual-branch collaborative prediction: the MHM branch and the HHN branch independently model candidate associations from two complementary perspectives, semantic propagation and local structure. (**D**) Sample-adaptive gating fusion and uncertainty-aware calibration: branch weights are dynamically assigned for each candidate association, and prediction divergence provides an empirical indicator of relative prediction risk. Predictions for high-divergence samples are adjusted toward a neutral reference value.

This work makes three contributions to CDA prediction. First, we combine hypergraph convolution with cross-view contrastive constraints to learn group-level representations across multiple circRNA and disease similarity views. Second, we combine semantic propagation and local structural signals in a dual-branch architecture. Third, we use divergence between branch predictions as a sample-level uncertainty indicator in fusion, and observe an AUC gap of 0.1263 between the highest- and lowest-confidence sample groups, supporting its empirical risk-stratification role. The overall framework of MHUCDA is illustrated in Figure 1.

## 2. Materials and Methods

### 2.1. Dataset

CDA data were obtained from four publicly available databases: CircR2Disease [29], CircAtlas v2.0 [30], Circ2Disease [31], and CircRNADisease [32]. Table 1 summarizes the dataset statistics. CircR2Disease was selected as the primary benchmark dataset based on its data reliability, coverage, and benchmark applicability [33]. This database includes both high-throughput sequencing records and experimentally validated associations. From this dataset, a CDA matrix was constructed as unified input for similarity computation, node representation learning, and model training.

Candidate negative samples were randomly drawn from unverified circRNA–disease pairs to match the number of positive samples, yielding a balanced 1:1 ratio. These pairs are not experimentally confirmed negative associations. To ensure reproducibility, a fixed random seed was applied during sampling. After 20% of the sampled positive and negative pairs had been reserved, the remaining balanced pool was used for five-fold cross-validation. For CircR2Disease, this pool comprised 486 positive and 486 candidate negative pairs. The folds were formed at the association-pair level, allowing the same circRNA or disease to occur in both training and evaluation partitions; this evaluates associations among known entities.

### 2.2. Construction of Similarity Representations

To characterize latent relationships between circRNAs and diseases, multiple similarity types are constructed from two complementary perspectives: association network topology and biological prior knowledge [34]. Let $A\in \{0,1{\}}^{n_{c}\times n_{d}}$ denote the CDA matrix, where $A_{ij}=1$ if a validated association exists between circRNA $c_{i}$ and disease $d_{j}$, and $A_{ij}=0$ otherwise.

#### 2.2.1. Disease Gaussian Interaction Similarity

The topology of the association network encodes latent similarity among disease nodes [35]. Diseases sharing similar circRNA association patterns are more likely to have related pathological mechanisms. Based on this assumption, disease Gaussian interaction similarity is constructed using interaction profile vectors derived from the association matrix [36]. For disease $d_{j}$, its interaction profile vector is defined as the j-th column of A:

(1) $$v\left(d_{j}\right)=A\left(:,j\right)$$

The Gaussian interaction similarity between diseases $d_{j}$ and $d_{j^{\prime }}$ is then defined as:

(2) $$S_{\mathrm{Gau}}^{d}\left(j,j^{\prime }\right)=exp\left(-\eta_{d}\cdot {∥v\left(d_{j}\right)-v\left(d_{j^{\prime }}\right)∥}^{2}\right)$$

where the bandwidth parameter $\eta_{d}$ is adaptively determined by the mean squared norm of all disease interaction profile vectors:

(3) $$\eta_{d}=\frac{n_{d}}{\sum_{j=1}^{n_{d}}{∥v\left(d_{j}\right)∥}^{2}}$$

This adaptive mechanism scales the similarity measure with the overall density of the association network, yielding the disease Gaussian interaction similarity matrix $S_{\mathrm{Gau}}^{d}$.

#### 2.2.2. circRNA Gaussian Interaction Similarity

Similarly, the interaction profile vector of circRNA $c_{i}$ is defined as the i-th row of A:

(4) $$v\left(c_{i}\right)=A\left(i,:\right)$$

CircRNAs with similar disease association patterns are more likely to share biological functions [37]. Based on this assumption, the Gaussian interaction similarity between circRNAs $c_{i}$ and $c_{i^{\prime }}$ is defined as:

(5) $$S_{\mathrm{Gau}}^{c}\left(i,i^{\prime }\right)=exp\left(-\eta_{c}\cdot {∥v\left(c_{i}\right)-v\left(c_{i^{\prime }}\right)∥}^{2}\right)$$

where the bandwidth parameter $\eta_{c}$ is likewise adaptively determined:

(6) $$\eta_{c}=\frac{n_{c}}{\sum_{i=1}^{n_{c}}{∥v\left(c_{i}\right)∥}^{2}}$$

This yields the circRNA Gaussian interaction similarity matrix $S_{\mathrm{Gau}}^{c}$.

#### 2.2.3. Disease Semantic Similarity

To incorporate biological prior knowledge independent of the association network structure, disease semantic similarity is constructed using the hierarchical structure of the Medical Subject Headings (MeSH) ontology [38]. In MeSH, each disease is organized within a directed acyclic graph (DAG) structure. The semantic graph corresponding to disease $d_{j}$ is defined as:

(7) $$\mathrm{DAG}\left(d_{j}\right)=\left(d_{j},N_{d_{j}},E_{d_{j}}\right)$$

where $N_{d_{j}}$ denotes the node set comprising $d_{j}$ itself and all its ancestor nodes, and $E_{d_{j}}$ denotes the corresponding set of directed edges. The semantic contribution value $D_{d_{j}}\left(t\right)$ of node $t\in N_{d_{j}}$ to the target disease $d_{j}$ is computed recursively as:

(8) $$D_{d_{j}}\left(t\right)=\left\{\begin{array}{ll} 1 & t=d_{j}\\ max\left\{\mu \cdot D_{d_{j}}\left(t^{\prime }\right)|t^{\prime }\in \mathrm{children}\;\mathrm{of}\;t\right\} & t\neq d_{j} \end{array}\right.$$

where μ=0.5 is the semantic decay factor governing propagation along hierarchical edges. The overall semantic value of disease $d_{j}$ is defined as the sum of contribution values across all nodes in its DAG:

(9) $$V\left(d_{j}\right)=\sum_{t\in N_{d_{j}}}D_{d_{j}}\left(t\right)$$

The semantic similarity between diseases $d_{j}$ and $d_{j^{\prime }}$ is obtained by quantifying the combined semantic contributions of their shared ancestor nodes, normalized by their respective overall semantic values:

(10) $$S_{\mathrm{Sem}}^{d}\left(j,j^{\prime }\right)=\frac{\sum_{t\in N_{d_{j}}\cap N_{d_{j^{\prime }}}}\left[D_{d_{j}}\left(t\right)+D_{d_{j^{\prime }}}\left(t\right)\right]}{\mathrm{DV}\left(d_{j}\right)+\mathrm{DV}\left(d_{j^{\prime }}\right)}$$

This yields the disease semantic similarity matrix $S_{\mathrm{Sem}}^{d}$, which captures biological disease relationships independently of association network topology.

#### 2.2.4. circRNA Functional Similarity

Beyond topological similarity, circRNA relationships are further characterized from a functional perspective. If the disease sets associated with two circRNAs are semantically similar, the two circRNAs are more likely to share biological functions or regulatory mechanisms [39]. Let $D\left(c_{i}\right)$ and $D\left(c_{i^{\prime }}\right)$ denote the sets of known associated diseases for circRNAs $c_{i}$ and $c_{i^{\prime }}$, respectively. Their functional similarity is defined as:

(11) $$S_{\mathrm{Fun}}^{c}\left(i,i^{\prime }\right)=\frac{\sum_{d_{p}\in D\left(c_{i}\right)}\underset{d_{q}\in D\left(c_{i^{\prime }}\right)}{max}S_{\mathrm{Sem}}^{d}\left(p,q\right)+\sum_{d_{q}\in D\left(c_{i^{\prime }}\right)}\underset{d_{p}\in D\left(c_{i}\right)}{max}S_{\mathrm{Sem}}^{d}\left(q,p\right)}{\left|D\left(c_{i}\right)\right|+\left|D\left(c_{i^{\prime }}\right)\right|}$$

This bidirectional maximum matching strategy provides a symmetric measure of the semantic alignment between the two disease sets, yielding the circRNA functional similarity matrix $S_{\mathrm{Fun}}^{c}$.

#### 2.2.5. Multi-Source Similarity Integration

Gaussian interaction similarity captures the topological features of the association network, whereas semantic and functional similarity encode complementary biological prior knowledge [40]. To combine these two information types, a conditional fusion strategy is adopted, constructing separate integrated similarity matrices for circRNAs and diseases. For circRNA nodes, functional similarity is used as the primary measure when it exists between $c_{i}$ and $c_{i^{\prime }}$; otherwise, Gaussian interaction similarity is used. The integrated similarity matrix $S_{\mathrm{Int}}^{c}$ is defined as:

(12) $$S_{\mathrm{Int}}^{c}\left(i,i^{\prime }\right)=\left\{\begin{array}{ll} S_{\mathrm{Fun}}^{c}\left(i,i^{\prime }\right) & \mathrm{if}\;\mathrm{functional}\;\mathrm{similarity}\;\mathrm{exists}\;\mathrm{between}\;c_{i}\;\mathrm{and}\;c_{i^{\prime }}\\ S_{\mathrm{Gau}}^{c}\left(i,i^{\prime }\right) & \mathrm{otherwise} \end{array}\right.$$

For disease nodes, semantic similarity serves as the primary measure when it exists between $d_{j}$ and $d_{j^{\prime }}$; otherwise, Gaussian interaction similarity is used. The integrated similarity matrix $S_{\mathrm{Int}}^{d}$ is defined as:

(13) $$S_{\mathrm{Int}}^{d}\left(j,j^{\prime }\right)=\left\{\begin{array}{ll} S_{\mathrm{Sem}}^{d}\left(j,j^{\prime }\right) & \mathrm{if}\;\mathrm{semantic}\;\mathrm{similarity}\;\mathrm{exists}\;\mathrm{between}\;d_{j}\;\mathrm{and}\;d_{j^{\prime }}\\ S_{\mathrm{Gau}}^{d}\left(j,j^{\prime }\right) & \mathrm{otherwise} \end{array}\right.$$

Through this integration, $S_{\mathrm{Int}}^{c}$ and $S_{\mathrm{Int}}^{d}$ retain the structural information of the association network while incorporating functional and semantic priors. Together with $S_{\mathrm{Gau}}^{c}$, $S_{\mathrm{Fun}}^{c}$, $S_{\mathrm{Gau}}^{d}$, and $S_{\mathrm{Sem}}^{d}$, these integrated matrices constitute three similarity views for each entity type, providing diverse inputs for the subsequent multi-view hypergraph contrastive embedding module.

### 2.3. Multi-View Hypergraph Contrastive Embedding

Hypergraph convolution represents groups of similar nodes through hyperedges, allowing joint information propagation among their members [41]. For each node, a hyperedge is constructed using a k-nearest neighbor (KNN) strategy, yielding the hypergraph incidence matrix $H\in \{0,1{\}}^{n\times n}$, where n denotes the number of nodes on the respective entity side. The normalized hypergraph Laplacian is then defined as:

(14) $$\Theta =D_{v}^{-\frac{1}{2}}HD_{e}^{-1}H^{⊤}D_{v}^{-\frac{1}{2}}$$

where $D_{v}$ and $D_{e}$ denote the node degree matrix and the hyperedge degree matrix, respectively. Given the node feature matrix X, the forward propagation of the hypergraph convolution layer is defined as:

(15) $$Z=\sigma \left(\mathrm{BN}\left(\Theta XW\right)\right)$$

In contrast to conventional graph convolution, which captures only pairwise interactions, hypergraph convolution propagates information jointly among all member nodes within a hyperedge via $HD_{e}^{-1}H^{⊤}$, explicitly capturing high-order cooperative relationships. For each entity type, three hypergraph convolutional encoders with independent parameters are instantiated, each encoding one of the three similarity views and producing view-specific representations $Z^{\left(1\right)},Z^{\left(2\right)}$, and $Z^{\left(3\right)}$.

Similarity views differ in their value distributions and topological density. In the absence of explicit alignment constraints, independently optimized encoders are prone to semantic drift, reducing the benefit of multi-view fusion. To address this, a cross-view InfoNCE contrastive loss is introduced, treating the embeddings of the same node across different views as positive pairs and all other nodes as negative samples:

(16) $$L_{CL}=-\frac{1}{N}\sum_{k=1}^{N}log\frac{exp\left(p_{k}^{\left(\alpha \right)}\cdot \frac{p_{k}^{\left(\beta \right)}}{\tau }\right)}{\sum_{l=1}^{N}exp\left(p_{k}^{\left(\alpha \right)}\cdot \frac{p_{l}^{\left(\beta \right)}}{\tau }\right)}$$

where $p_{k}^{\left(\alpha \right)}$ denotes the L2-normalized projection of node k in view α, τ is the temperature hyperparameter, and N denotes the total number of nodes on the respective entity side. The total contrastive loss is computed independently for circRNAs and diseases and subsequently summed: $L_{CL}=L_{CL}^{c}+L_{CL}^{d}$. This cross-view contrastive objective serves as a node-level self-supervised signal, requiring no association-level labels during optimization.

Similarity matrices are precomputed from the complete association matrix before cross-validation partitioning. The supervised training objective uses association labels from the training partition of each fold. After cross-view alignment, embeddings from the three views are concatenated and compressed to a common dimension through a fusion network.

In the reconstruction comparison, Gaussian interaction profile similarities for circRNAs and diseases and circRNA functional similarity were recomputed using only the positive training associations in each cross-validation fold. The graph relations and hypergraphs that directly depend on these three similarity matrices were updated accordingly.

### 2.4. Dual-Branch Parallel Collaborative Prediction

Semantic propagation and local structural signals provide complementary information: the former capture indirect associations through shared neighbors, while the latter describe local features of candidate pairs [9]. The MHM and HHN branches therefore model candidate associations through separate prediction pathways.

The MHM branch takes $E_{c}$ and $E_{d}$ as initial node features and defines several meta-paths of the “similarity–association–similarity” type on the heterogeneous graph. GIN is applied to perform aggregation along each meta-path:

(17) $$z_{v}^{\Phi_{k}}={MLP}^{\Phi_{k}}\left(\left(1+\varepsilon^{\Phi_{k}}\right)\cdot h_{v}+\sum_{u\in N_{\Phi_{k}}\left(v\right)}h_{u}\right)$$

where $N_{\Phi_{k}}\left(v\right)$ denotes the set of reachable neighbors of node v along meta-path $\Phi_{k}$, and parameters are not shared across different meta-paths. GIN achieves the theoretical upper bound of aggregation expressiveness in the Weisfeiler–Leman graph isomorphism test, making it particularly effective for semantic extraction from structurally diverse meta-paths. The outputs of all meta-paths are aggregated through a two-level hierarchical semantic attention mechanism. Bidirectional collaborative cross-attention is then applied to enable information exchange between circRNA and disease nodes. The prediction score for a candidate pair $\left(c_{i},d_{j}\right)$ is then obtained via a Hadamard product followed by an MLP:

(18) $${\hat{y}}_{MHM}=\sigma \left(MLP\left(h_{c_{i}}^{co}⊙h_{d_{j}}^{co}\right)\right)\in \left(0,1\right)$$

The HHN branch models candidate-pair features through local structural processing. It first performs two-layer GCN aggregation on the circRNA–disease bipartite graph, then forms a six-node local graph using the two candidate-entity embeddings and four learnable virtual nodes. A deep GCN with residual connections extracts features from this graph:

(19) $$F^{\left(l+1\right)}=F^{\left(l\right)}+\sigma \left({\tilde{D}}^{-\frac{1}{2}}L{\tilde{D}}^{-\frac{1}{2}}F^{\left(l\right)}W^{\left(l\right)}\right)$$

where L denotes the normalized adjacency matrix of the local subgraph and $\tilde{D}$ denotes the corresponding degree matrix. Node representations from successive layers are aggregated using multi-head attention. The prediction score is then obtained through a Hadamard product and an MLP:

(20) $${\hat{y}}_{HHN}=\sigma \left(MLP\left(r_{c_{i}}⊙r_{d_{j}}\right)\right)\in \left(0,1\right)$$

The two branches independently yield ${\hat{y}}_{MHM}$ and ${\hat{y}}_{HHN}$, which encode semantic propagation and local structural signals, respectively. The subsequent module combines these predictions adaptively for each sample.

### 2.5. Sample-Adaptive Gating Fusion and Uncertainty-Aware Calibration

The relative contribution of the two branches may vary across candidate associations. Local structural signals may be informative for circRNAs with abundant association records, whereas semantic propagation may provide complementary information when direct associations are sparse [42]. This sample-level heterogeneity requires the fusion strategy to address two distinct levels: branch weights should be sample-adaptive rather than globally fixed, and when the two branches diverge substantially, their disagreement can provide additional information for candidate risk ranking.

Level 1: Sample-adaptive gating fusion. For a candidate pair $\left(c_{i},d_{j}\right)$, the node embeddings $h_{c_{i}}$ and $h_{d_{j}}$ are extracted from $E_{c}$ and $E_{d}$. Together with the dual-branch prediction scores, these embeddings are passed to the gating network to compute the sample-level fusion weight:

(21) $$g=\sigma \left(W_{2}\cdot \mathrm{ReLU}\left(W_{1}\left[h_{c_{i}}{∥h}_{d_{j}}∥{\hat{y}}_{MHM}∥{\hat{y}}_{HHN}\right]+b_{1}\right)+b_{2}\right)$$

The gating weight $g\in \left(0,1\right)$ is computed independently for each candidate pair, allowing per-sample adaptation of the relative contribution of each signal. The dual-branch predictions are then combined via weighted fusion:

(22) $${\hat{y}}_{\mathrm{fus}}=g\cdot {\hat{y}}_{MHM}+\left(1-g\right)\cdot {\hat{y}}_{HHN}$$

Level 2: Uncertainty-aware calibration. Gating fusion combines the branch predictions, while their absolute difference is used as an empirical indicator of relative prediction risk:

(23) $$u=\left|{\hat{y}}_{MHM}-{\hat{y}}_{HHN}\right|$$

The fused prediction is then adjusted based on the confidence score conf=1−u:

(24) $$\hat{y}=\mathrm{conf}\cdot {\hat{y}}_{fus}+\left(1-\mathrm{conf}\right)\cdot 0.5$$

The value 0.5 is a score-adjustment reference point for the balanced evaluation setting used in this study. For binary classification, the entropy of the Bernoulli distribution, $H\left(p\right)=-plogp-\left(1-p\right)log\left(1-p\right)$, attains its unique maximum at p=0.5. This reference value does not estimate the prevalence of true CDAs. When the two branches diverge substantially, predictions are shifted toward 0.5, reducing the extremity of the scores. Conversely, when the two branches are in high agreement, conf≈1 and the final prediction remains close to the fused result. As an empirical risk indicator, u can support relative risk ranking of candidate associations and help prioritize experimental validation. For model training, Focal Loss is adopted as the primary loss function to mitigate the class imbalance between positive and negative samples. Combined with the cross-view contrastive loss, the total loss function is defined as:

(25) $$L_{\mathrm{total}}=L_{\mathrm{focal}}+\lambda \cdot L_{\mathrm{CL}}$$

where λ is the weighting coefficient balancing the contributions of the primary prediction task and multi-view representation learning.

### 2.6. Implementation Details and Computational Cost

The implementation uses Adam with a batch size of 128 and weight decay of 10^−4^. The base learning rates are 0.0007 for the main parameter group and 0.00084 for the multi-view embedding modules, with 30 epochs of linear warmup followed by cosine annealing. Training runs for at most 250 epochs, with AUC-based early stopping and a patience of 150 epochs. After the first 180 epochs, model states are collected every four epochs for stochastic weight averaging (SWA); the averaged candidate is retained only if its AUC exceeds that of the best individual checkpoint. Checkpoint selection, early stopping and SWA selection use the same fold subsequently used for evaluation. The circRNA and disease projection heads each use a 128→128→64 architecture with an intermediate ReLU and L2-normalized outputs.

The supplementary cold-start experiments separated model selection from testing. All target test entities were excluded from both the development training and validation sets. Model configurations and fusion strategies were selected using the development validation results. During the final entity-wise leave-one-out evaluation, checkpoint selection, early stopping and SWA selection used a separate validation set. The selected checkpoint was then loaded directly for testing, without using test data for model selection.

On an Intel Core i9-13900H CPU with four computational threads, a 250-epoch run of the first fold, including model selection, took 436.83 s and reached a sampled peak process memory of 1009.05 MiB, excluding one-time input and graph preparation. With the model, input graph and batch size fixed, mean inference time increased from 0.1417 s for 256 candidates to 9.1507 s for 16,384 candidates, while sampled peak process memory remained at 770–778 MiB. Inference times were averaged over five repetitions after warmup and excluded model loading and input preparation.

HHN forms a six-node local graph from the two candidate-entity embeddings and four learnable virtual nodes. For fixed depth and attention configuration, the local computation per pair is O(k^3^ + k^2^d + kd^2^), where k is the local node count and d is the feature dimension. With k = 6 and d = 128, this local computation scales as O(N) for N candidate pairs.

## 3. Results

### 3.1. Evaluation Metrics

Five-fold cross-validation is performed on the CircR2Disease benchmark dataset, and six metrics are used for evaluation: AUC, AUPR, F1-score, Precision, Recall, and Accuracy. AUC and AUPR are threshold-independent, reflecting overall discrimination and positive-class ranking, respectively. The originally reported F1-score, Precision, Recall, and Accuracy use the threshold that maximizes F1-score on each evaluation fold. This is a post-hoc threshold choice using evaluation labels and can yield optimistic threshold-dependent estimates. We therefore also report a fixed-threshold analysis at 0.5 using the same saved predictions. All metrics are reported as the mean and population standard deviation across the five folds. These metrics are defined as follows:

(26) $$\mathrm{Precision}\;=\;\frac{\mathrm{TP}}{\mathrm{TP}\;+\;\mathrm{FP}}$$

(27) $$\mathrm{Recall}=\frac{\mathrm{TP}}{\mathrm{TP}+\mathrm{FN}}$$

(28) $$F1=\frac{2\;\times \;\mathrm{Precision}\;\times \;\mathrm{Recall}}{\mathrm{Precision}+\mathrm{Recall}}$$

### 3.2. Performance Evaluation

**Figure 2:**
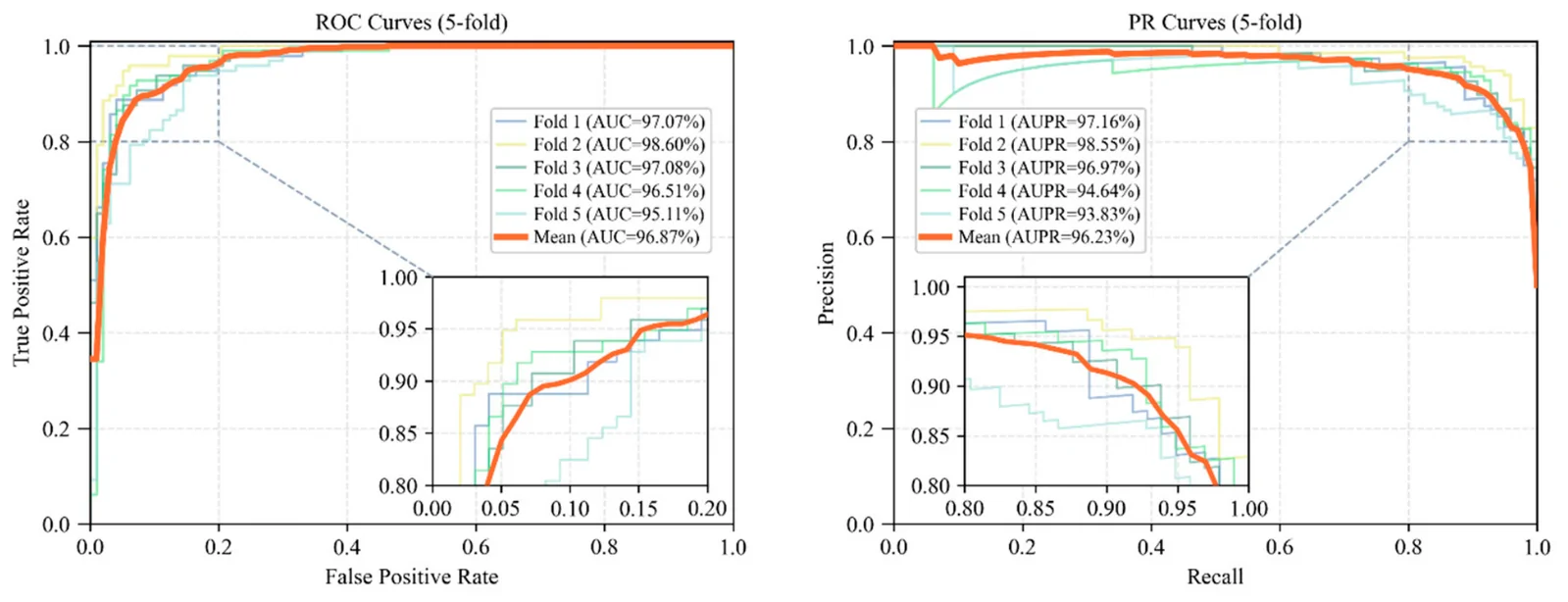
ROC curves and PR curves of MHUCDA under five-fold cross-validation.

Table 2 presents the five-fold cross-validation results, and Figure 2 shows the corresponding ROC and PR curves. MHUCDA achieves a mean AUC of 0.9687 and a mean AUPR of 0.9623 with limited variation across the five folds. Even in test folds with relatively sparse association records, the model maintains strong positive class identification performance. These results demonstrate that MHUCDA effectively captures latent CDAs under data-sparse conditions.

Using the original saved predictions with a fixed classification threshold of 0.5, the five-fold F1-score, Precision, Recall and Accuracy are 0.8902 ± 0.0162, 0.8274 ± 0.0331, 0.9651 ± 0.0229 and 0.8806 ± 0.0201, respectively. AUC and AUPR remain 0.9687 and 0.9623 because the continuous scores are unchanged.

After reconstruction, the five-fold mean AUC decreased from 0.9687 to 0.9089 and AUPR from 0.9623 to 0.8981. Fewer available associations within each training fold and changes in neighborhood relationships may affect the extraction of association features. These differences suggest that complete association information may contribute to more favorable performance estimates under the original setting. Both metrics remained close to 0.90 after reconstruction, indicating that the model retained an ability to identify and rank candidate associations.

Ten random negative-sampling evaluations were conducted using the original saved five-fold models. AUC, AUPR and their variability are reported in Table S1.

### 3.3. Multi-Dataset Validation and Confidence Analysis

#### 3.3.1. Performance Across Four Datasets

MHUCDA was trained and evaluated separately within CircR2Disease, CircAtlas 2.0, Circ2Disease and CircRNADisease, using each database’s own inputs and five-fold partitions. A new model and optimizer were initialized for each fold, and trained parameters were not transferred between databases. As shown in Table 3, AUC exceeds 0.9590 and AUPR exceeds 0.9500 in all four datasets. These results indicate consistent performance in separate within-dataset evaluations. The databases contain overlapping positive associations, which may arise from shared source literature or resource integration.

#### 3.3.2. Uncertainty Stratification Analysis

**Figure 3:**
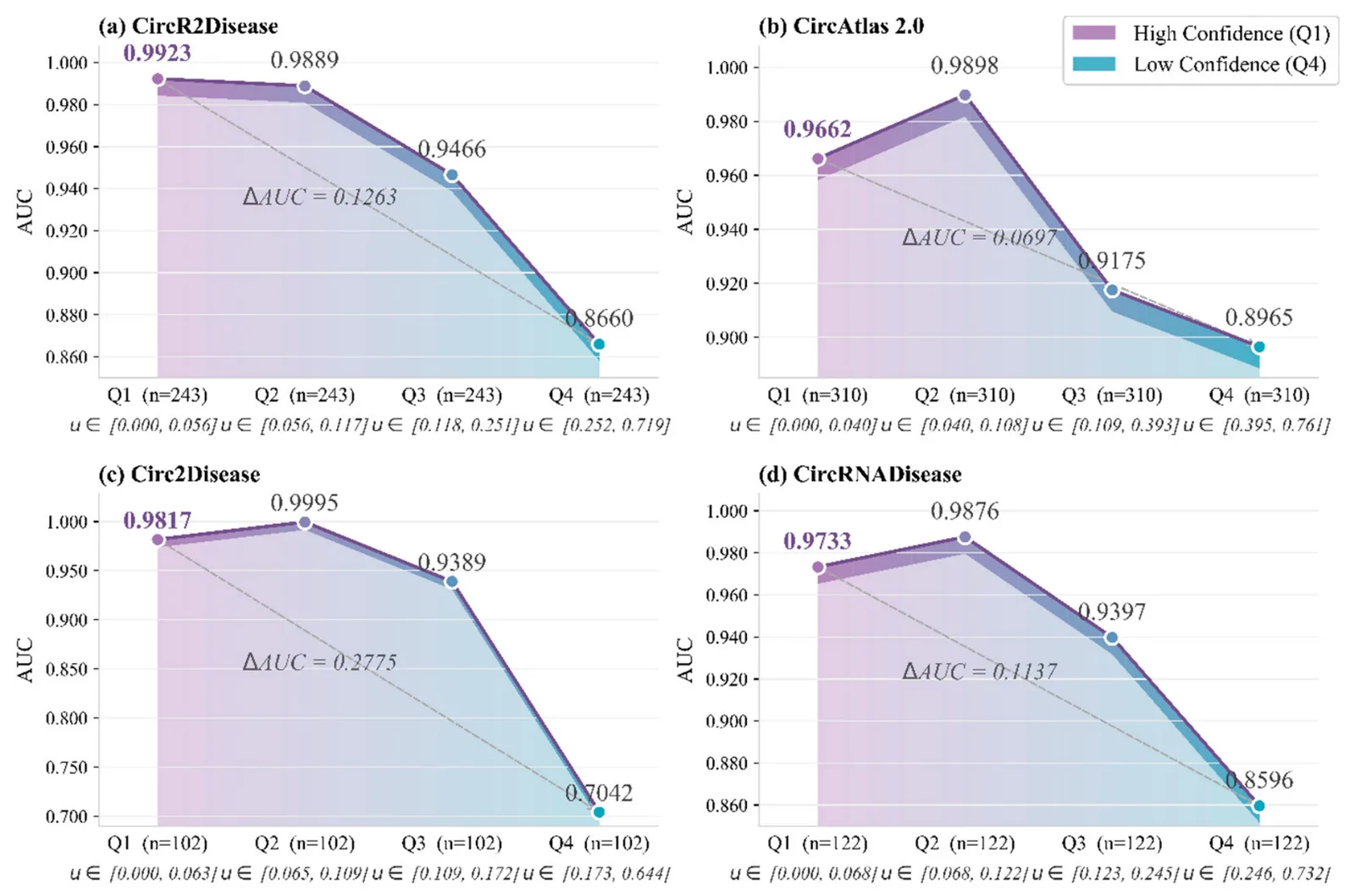
Predictive confidence analysis results under uncertainty stratification. (**a**) Confidence-Stratified AUC on CircR2Disease (**b**) Confidence-Stratified AUC on CircAtlas 2.0 (**c**) Confidence-Stratified AUC on Circ2Disease (**d**) Confidence-Stratified AUC on CircRNADisease.

Test samples from each dataset were equally divided into four groups (Q1 to Q4) in ascending order of dual-branch prediction divergence u. The correspondence between confidence ranking and actual predictive performance was then examined. As shown in Figure 3, all four datasets exhibit pronounced performance stratification between the high-confidence groups (Q1/Q2) and the low-confidence groups (Q3/Q4). The AUC gaps between the highest-confidence group (Q1) and the lowest-confidence group (Q4) across the four datasets are 0.1263, 0.0723, 0.2775, and 0.1137, respectively. Minor fluctuations between Q1 and Q2, and between Q3 and Q4, are observed in certain datasets. These fluctuations may reflect sampling variation and indicate that performance need not change monotonically between adjacent groups. Using a fixed classification threshold of 0.5, the original five-fold predictions yielded an error-detection AUROC of 0.7570 ± 0.0705 for divergence. Retaining approximately 50% of samples in ascending divergence order reduced the mean error rate from 11.94% to 4.12%. The mean error-detection AUROCs for the entropy of the gated and adjusted outputs were 0.7067 and 0.8390, respectively. These results support an empirical risk-ranking role for branch divergence, with adjusted-output entropy performing best in this comparison.

#### 3.3.3. Confidence-Guided Candidate Prioritization

The Q1 group, comprising the top 25% of samples with the highest confidence scores (conf=1−u), is examined in more detail. As reported in Table 4, the Q1 group achieves superior AUC, AUPR, F1-score, and Recall relative to the full test set across all four datasets, with average improvements of +0.0150 in AUC, +0.0472 in F1-score, and +0.0589 in Recall.

The minor Precision decline in the Circ2Disease Q1 group (−0.0097) may reflect sampling variation or threshold sensitivity under the small-sample condition (n=102); its AUPR of 0.9724 nevertheless indicates strong positive-class ranking performance in this subset. For practical scenarios with limited experimental validation resources, confidence ranking provides a quantitative basis for prioritizing candidate associations without requiring additional supervision.

### 3.4. Hyperparameter Sensitivity Analysis

**Figure 4:**
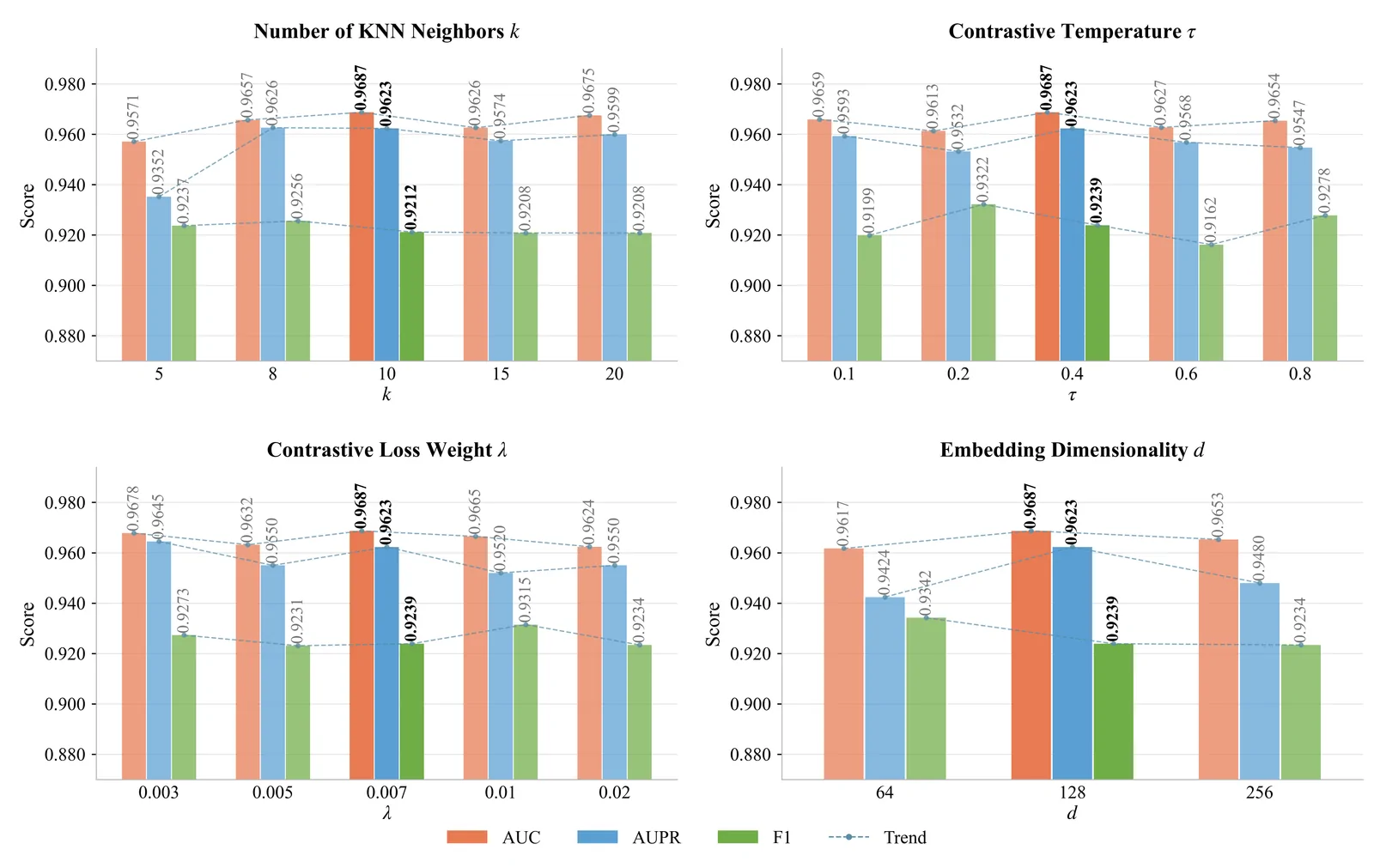
Hyperparameter sensitivity analysis of MHUCDA.

To assess the robustness of MHUCDA to hyperparameter selection, a systematic sweep was conducted over four key hyperparameters: the number of KNN neighbors K, the contrastive temperature τ, the contrastive loss weighting coefficient λ, and the embedding dimensionality d. All remaining parameters were held at their default values. As shown in Figure 4, the model achieves optimal performance at K=10, τ=0.4, λ=0.007, and d=128.

An excessively small K limits the high-order neighborhood coverage of the hypergraph, whereas an excessively large K introduces noisy edges; both cases impair feature extraction in hypergraph convolution. Although the three similarity views exhibit heterogeneous topological densities and value distributions, KNN-based hyperedge construction operates through relative ranking rather than absolute thresholds. This allows a uniform K to consistently capture the high-order neighborhood structure across all views without requiring view-specific parameter tuning. The choice of τ directly governs the optimization behavior of contrastive learning: an excessively small τ renders the loss function overly sharp, leading to training instability, while an excessively large τ weakens discriminative capability across views. The weighting coefficient λ must maintain a balance between contrastive regularization and primary task learning; deviations in either direction disrupt this balance and degrade performance. An excessively small d restricts node representation capacity, whereas an excessively large d introduces redundant parameters and increases the risk of overfitting. Performance varies little near each optimum, indicating stability within the tested parameter ranges.

### 3.5. Ablation Study: Embedding Module

**Figure 5:**
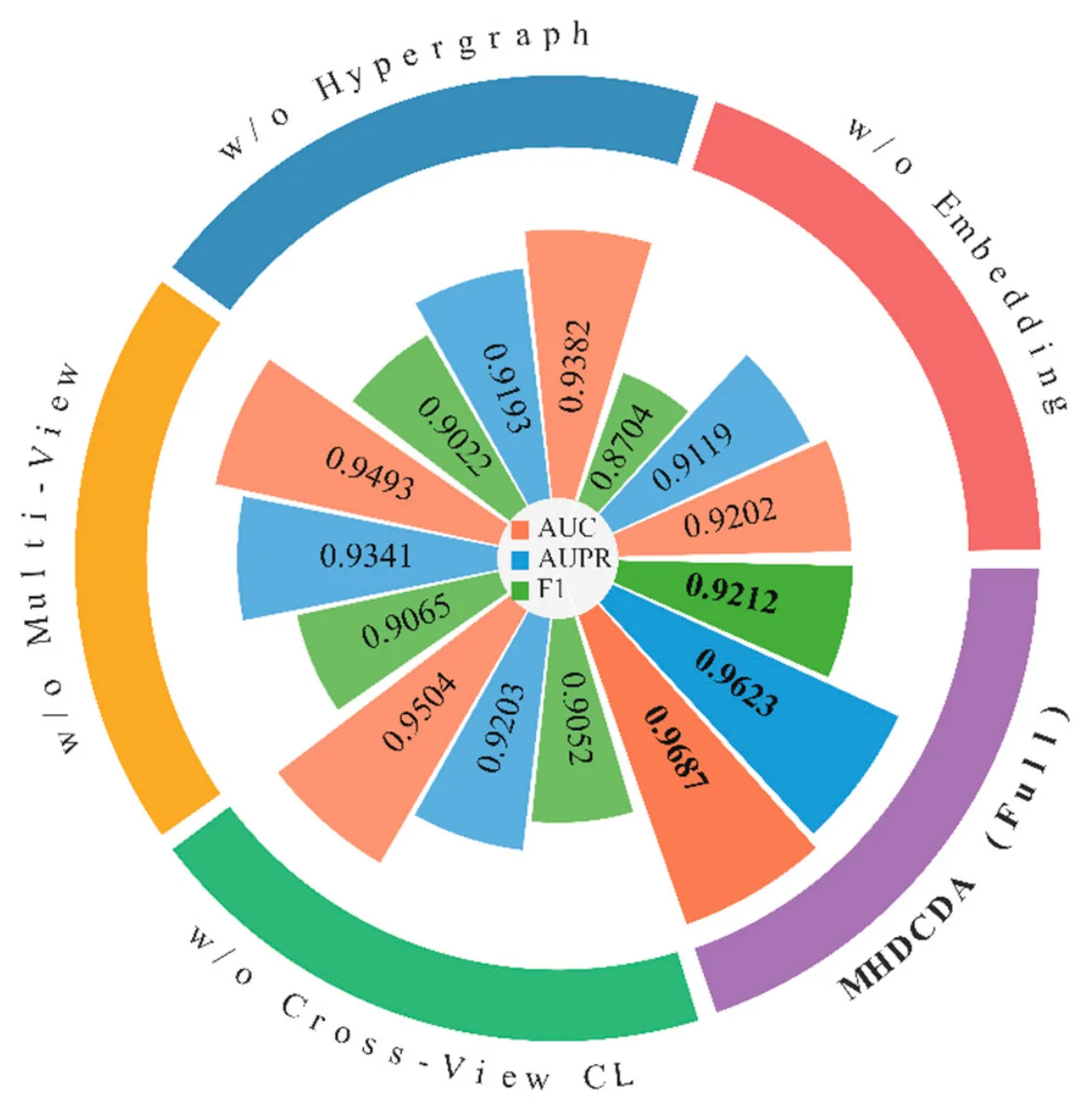
Performance comparison of ablation variants for multi-view hypergraph contrastive embedding.

To assess the contribution of each component in the multi-view hypergraph contrastive embedding module, four ablation variants are evaluated: replacing hypergraph convolution with conventional GCN (w/o Hypergraph), retaining only a single similarity view (w/o Multi-View), removing the cross-view contrastive loss (w/o Cross-View CL), and removing the entire embedding module (w/o Embedding). As shown in Figure 5, removing the embedding module reduces AUC to 0.9202, supporting the contribution of the learned multi-view representations to association prediction.

Replacing hypergraph convolution with conventional GCN yields an AUC of 0.9382 and an AUPR of 0.9193, compared with 0.9687 and 0.9623 for the complete model, respectively. The differences of 3.05 and 4.30 percentage points support the use of hypergraph representations in this setting. Using a single view or removing the cross-view contrastive loss also reduces AUC and AUPR relative to the complete model. Together, these comparisons support the contributions of multi-view information and cross-view alignment to association discrimination and ranking.

### 3.6. Ablation Study: Dual-Branch Architecture and Fusion Strategy

**Figure 6:**
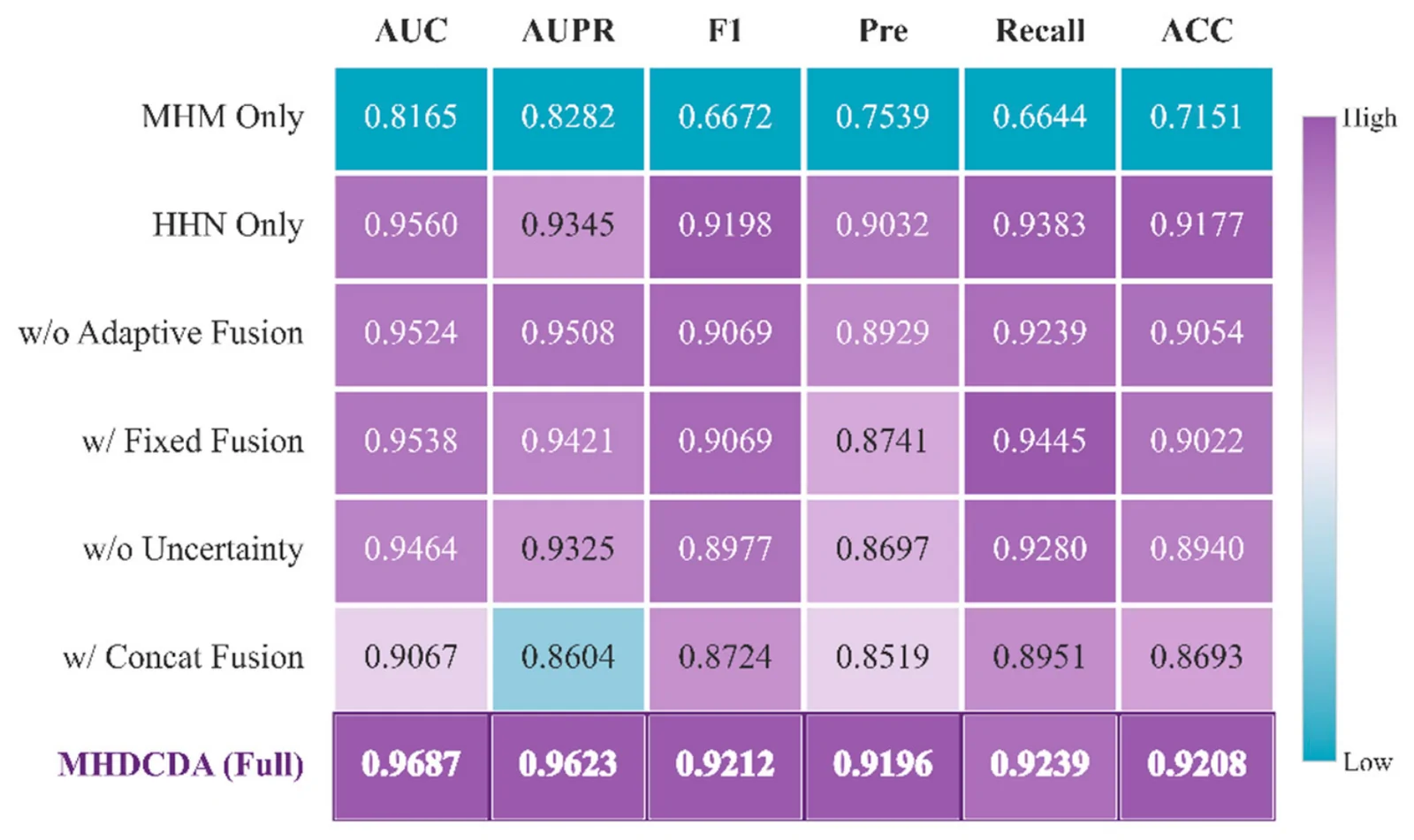
Performance comparison of ablation variants for dual-branch prediction and sample-adaptive gating fusion.

To assess the contribution of each component in the dual-branch collaborative prediction and sample-adaptive gating fusion modules, six ablation variants are evaluated: retaining only the MHM semantic branch (MHM only), retaining only the HHN structural branch (HHN only), removing adaptive fusion and directly merging the dual-branch outputs (w/o Adaptive Fusion), replacing the adaptive gating mechanism with fixed-weight fusion (w/ Fixed Fusion), removing uncertainty-aware calibration (w/o Uncertainty), and replacing gating fusion with concatenation-based fusion (w/ Concat Fusion). The experimental results are presented in Figure 6.

At the branch level, MHM only achieves an AUC of 0.8165 and an AUPR of 0.8282, while HHN only achieves 0.9560 and 0.9345, respectively. The complete model reaches 0.9687 and 0.9623, exceeding both single-branch variants. These results support combining the semantic propagation and local structural signals.

At the fusion level, both w/o Adaptive Fusion and w/ Fixed Fusion yield lower AUC and AUPR than the complete model. Removing uncertainty-aware calibration also lowers both ranking metrics. Concatenation-based fusion has the lowest AUC and AUPR among the fusion variants, at 0.9067 and 0.8604, respectively. These comparisons support the contributions of sample-adaptive fusion and divergence-based score adjustment.

The original predictions were evaluated at seven ratios of total positive to negative class weights, ranging from 1:1 to 1:50. The output after uncertainty-aware calibration had higher AUPR than gated fusion alone at each ratio (Table S2).

A separate comparison of two outputs from the same five saved models shows that applying equation (24) increases the mean AUC from 0.9444 to 0.9687 and AUPR from 0.9111 to 0.9623. The corresponding ECE changes from 0.1208 to 0.1809, NLL from 0.3513 to 0.3662, and Brier score from 0.0997 to 0.1042. Thus, the observed benefit is in association ranking rather than improved probability calibration under the balanced evaluation setting.

### 3.7. Comparison with State-of-the-Art Methods

MHUCDA is compared with eight CDA prediction methods on the CircR2Disease benchmark. iCircDA-NEAE combines attribute network embedding with a dynamic convolutional autoencoder [43], while LGFRCDA uses LightGBM-based feature selection and a Transformer [44]. HGECDA learns heterogeneous network embeddings through meta-path-based random walks [45], and HGCNCDA employs high-order graph convolution [46]. MAGCDA uses multi-hop attention [47], whereas LMGATCDA combines a labeling trick with graph neural networks [48]. RPMVCDA integrates random perturbation with multi-view graph convolution [15], and MCHG combines multi-view hypergraph modeling with contrastive learning [49]. Baseline values are taken from the original papers to preserve the authors’ implementations and experimental configurations. Table 5 summarizes AUC, AUPR, ACC and F1.

MHUCDA achieves an AUC of 0.9687 and an AUPR of 0.9623, the highest reported values for these metrics in Table 5. Compared with RPMVCDA, the differences are 1.69 and 0.54 percentage points; compared with MCHG, they are 2.93 and 0.21 percentage points. Considering all four metrics, MHUCDA shows competitive overall performance.

### 3.8. Case Study

To evaluate the practical utility of MHUCDA in guiding experimental prioritization, all unverified candidate associations were ranked according to their prediction scores. The top 25 high-confidence candidates are listed in Table 6, among which 22 were supported by published literature, corresponding to a confirmation rate of 88%. These candidates were selected on the basis of both high prediction scores and low prediction uncertainty. Low branch divergence is used as a secondary criterion for candidate prioritization alongside the prediction score. To illustrate the correspondence between model-derived confidence scores and biologically interpretable associations, two candidates with independently validated ceRNA regulatory axes were selected for detailed analysis. The molecular mechanisms discussed below are derived entirely from independently published experimental studies.

Case A: hsa_circ_0003570 and hepatocellular carcinoma. hsa_circ_0003570 is derived from the FAM53B gene locus and is consistently downregulated in HCC tissues, with low expression being significantly associated with advanced tumor stage and poor prognosis [50]. MHUCDA assigned this association a high-confidence prediction. Mechanistically, hsa_circ_0003570 competitively binds the oncogenic hsa-miR-182-5p, thereby derepressing the tumor suppressor gene STARD13 and inhibiting HCC cell proliferation, migration, and invasion. The circ_0003570/miR-182-5p/STARD13 axis has been validated by dual-luciferase reporter assays and in vivo xenograft experiments. This case shows that the model’s high-confidence prediction corresponds to a well-characterized ceRNA regulatory axis, indicating that dual-branch prediction consistency captures genuine biological signals in this instance.

Case B: hsa_circ_0001313 and colorectal cancer. hsa_circ_0001313, also known as circCCDC66, is generated by back-splicing of exons 8–10 of the CCDC66 gene and functions as an oncogenic driver. It is upregulated in 88% of CRC patients and is associated with reduced overall survival [51]. MHUCDA likewise assigned this association a high-confidence prediction. circCCDC66 promotes CRC progression through two parallel ceRNA axes: it sponges hsa-miR-338-3p to mediate radiotherapy resistance and promote colony formation, and sponges hsa-miR-370 to upregulate MDM4 and drive proliferation and invasion. Both axes have been independently verified by functional assays. Unlike the tumor-suppressive circRNA in Case A, circCCDC66 has an oncogenic effect, yet both associations receive high-confidence predictions. This suggests that the uncertainty framework captures biological signals independently of the functional direction of the circRNA. Together, these two illustrative examples show that high-confidence predictions generated by MHUCDA correspond to associations with coherent molecular underpinnings, supporting the use of model-derived confidence scores as a quantitative basis for prioritizing experimental validation.

## 4. Discussion

On CircR2Disease, MHUCDA achieves an AUC of 0.9687 and an AUPR of 0.9623. Separate training and within-dataset evaluation on three additional databases also yield strong ranking performance. The AUC gap of 0.1263 between the highest- and lowest-confidence groups supports performance stratification by branch divergence, while the error-detection analysis provides more direct evidence of its empirical risk-ranking role. Case analyses further show that high-confidence predictions correspond to associations with experimentally verified ceRNA regulatory axes: hsa_circ_0003570 suppresses HCC progression through the miR-182-5p/STARD13 axis, whereas hsa_circ_0001313 promotes CRC progression through parallel miR-338-3p and miR-370 axes. Both associations rank among the model’s highest-confidence predictions.Several limitations remain. Representation learning depends on association-derived inputs, which can limit representation quality for circRNAs with sparse association records. When both branches are affected by the same systematic bias, their agreement may fail to reveal prediction errors, leading to overestimated confidence. In addition, unverified candidate negatives may include unknown positive associations, and the balanced evaluation pool does not represent the positive prevalence in the full candidate space. Association-pair cross-validation does not directly establish performance on unseen entities; withholding their associations reduces the interaction-profile and neighborhood evidence available to both branches. In the original five-fold experiments, the evaluation fold was used for model selection, which may favor models adapted to that fold and lead to more favorable performance estimates. The supplementary entity-wise leave-one-out cold-start evaluation covered five circRNAs and five diseases with relatively high association counts. AUC and AUPR for both branches, gated fusion, and the final output are reported in Table S3. Future work will pursue two directions that address these limitations. Incorporating pretrained RNA sequence encoders as auxiliary node features may reduce dependence on association density and improve representations of sparsely annotated circRNAs [52]. Constructing a multi-omics heterogeneous network that integrates miRNA and lncRNA nodes could support cross-entity consistency checks to identify shared biases and provide additional evidence for assessing prediction uncertainty.

## 5. Conclusions

MHUCDA integrates multi-view hypergraph contrastive embedding, dual-branch collaborative prediction, and sample-adaptive gating fusion for CDA prediction. Multi-view embedding provides node representations, while the two prediction branches capture semantic and local structural signals. Uncertainty-aware calibration uses branch divergence to adjust the fused prediction score. The resulting scores support association ranking, and branch divergence provides an empirical signal for candidate risk stratification. Together, these functions support the prioritization of candidate associations for experimental validation.

## Figures and Tables

**Table 1:** Statistics of the collected CDA datasets.

Database	CircRNAs	Diseases	Associations
CircR2Disease	561	100	607
CircAtlas	798	117	775
Circ2Disease	234	60	254
CircRNADisease	286	48	304

**Table 2:** Five-fold cross-validation results of MHUCDA. Threshold-dependent metrics use evaluation-fold maximum-F1 thresholds.

Fold	AUC	AUPR	F1	Precision	Recall	ACC
1	0.9707	0.9716	0.9206	0.9560	0.8878	0.9231
2	0.9860	0.9855	0.9436	0.9388	0.9485	0.9436
3	0.9708	0.9697	0.9192	0.9010	0.9381	0.9175
4	0.9651	0.9464	0.9271	0.9368	0.9175	0.9278
5	0.9511	0.9383	0.8955	0.8654	0.9278	0.8918
Mean	0.9687	0.9623	0.9212	0.9196	0.9239	0.9208
SD	0.0112	0.0174	0.0155	0.0325	0.0208	0.0169

**Table 3:** Performance of MHUCDA across multiple datasets. Threshold-dependent metrics use evaluation-fold maximum-F1 thresholds.

Database	AUC	AUPR	F1	Precision	Recall	ACC
CircAtlas 2.0	0.9627	0.9599	0.9220	0.9096	0.9371	0.9202
Circ2Disease	0.9593	0.9510	0.9113	0.9208	0.9073	0.9119
CircRNADisease	0.9629	0.9506	0.9192	0.9010	0.9381	0.9160
CircR2Disease	0.9687	0.9623	0.9212	0.9196	0.9239	0.9208

**Table 4:** Metric comparison between the Q1 group and the full test set. Threshold-dependent metrics use evaluation-fold maximum-F1 thresholds.

Dataset	Group	*n*	AUC	AUPR	F1	Recall	Precision	ACC
CircR2Disease	Q1	243	0.9923	0.9917	0.9721	0.9919	0.9531	0.9712
	Δ	—	+0.0236	+0.0294	+0.0509	+0.0680	+0.0335	+0.0504
CircAtlas 2.0	Q1	310	0.9662	0.9832	0.9736	0.9910	0.9567	0.9613
	Δ	—	+0.0035	+0.0233	+0.0516	+0.0539	+0.0471	+0.0411
Circ2Disease	Q1	102	0.9817	0.9724	0.9425	0.9762	0.9111	0.9510
	Δ	—	+0.0224	+0.0214	+0.0312	+0.0689	−0.0097	+0.0391
CircRNADisease	Q1	122	0.9733	0.9587	0.9744	0.9828	0.9661	0.9754
	Δ	—	+0.0104	+0.0081	+0.0552	+0.0447	+0.0651	+0.0594

Δ denotes the difference in each metric between the Q1 group and the full test set (Q1 − full test set), and “—” indicates that the entry is not applicable.

**Table 5:** Literature-reported comparison of MHUCDA and existing CDA prediction methods. MHUCDA ACC and F1 use evaluation-fold maximum-F1 thresholds; — denotes an unavailable value.

Method	AUC	AUPR	ACC	F1
iCircDA-NEAE	0.9258	—	0.9273	0.8939
LGFRCDA	0.9336	0.9330	0.8969	0.9009
HGECDA	0.9032	0.9123	0.8582	0.8523
HGCNCDA	0.9491	0.5302	0.9920	0.5532
MAGCDA	0.9323	—	0.9258	0.9264
LMGATCDA	0.9425	—	0.9537	0.8423
RPMVCDA	0.9518	0.9569	0.9378	0.9110
MCHG	0.9394	0.9602	0.9354	0.9322
MHUCDA	0.9687	0.9623	0.9208	0.9212

“—” indicates that the entry is not applicable.

**Table 6:** Literature validation of the top 25 high-confidence CDA candidates.

Rank	circRNA	Disease	Evidence
1	hsa_circ_0004136	Acute myeloid leukemia	33853086
2	hsa_circ_0000554	Esophageal cancer	33470617
3	hsa_circ_0003575	Atherosclerosis	37599528
4	hsa_circ_0002024	Bladder cancer	30972190
5	circ-Foxo3	Breast cancer	37400900
6	hsa_circ_0068033	Breast cancer	32184627
7	hsa_circ_0001667	Breast cancer	36811283
8	hsa_circ_0018289	Cervical cancer	35312113
9	hsa_circ_0001313	Colorectal cancer	35069793
10	hsa_circ_0000284	Diabetes mellitus	30286957
11	hsa_circ_0001313	Cervical cancer	Unconfirmed
12	hsa_circ_0081108	Diabetic retinopathy	32497630
13	hsa_circ_0109046	Endometrial cancer	33173333
14	hsa_circ_0007534	Glioma	Unconfirmed
15	hsa_circ_0037911	Essential hypertension	30861600
16	hsa_circ_0003570	Hepatocellular carcinoma	36241975
17	hsa_circ_0085154	Hepatocellular carcinoma	30655779
18	hsa_circ_0001821	Lung cancer	31484581
19	circRNA_002581	Nonalcoholic steatohepatitis	32054840
20	hsa_circ_0005567	Osteoarthritis	36278814
21	hsa_circ_0092509	Osteosarcoma	30338784
22	Circ_STXBP5	Glioma	Unconfirmed
23	hsa_circ_005239	Triple negative breast cancer	34668628
24	hsa_circ_0001204	Tuberculosis	36048566
25	hsa_circ_0054633	Type 2 diabetes mellitus	34272768

## Data Availability

Data Availability Statement: All code and data are publicly available in the GitHub repository: https://github.com/yhzhou165-netizen/MHUCDA (accessed on 2 November 2026).

## Supplementary Materials

The following supporting information can be downloaded at https://www.mdpi.com/article/10.3390/biology15212013/s1. Table S1: Repeated negative-sampling evaluation; Table S2: Class-ratio reweighting analysis; Table S3: Cold-start prediction results for circRNAs and diseases.
