# Supplementary material for: Multi-View Hypergraph Contrastive Learning for Uncertainty-Aware circRNA–Disease Association Prediction
Source: Biology (Basel). 2026 Nov 5;15(21):2013. doi: 10.3390/biology15212013 (PMC13761884; doi:10.3390/biology15212013)
Supplement: Supplementary file 1 [file biology-15-02013-s001.zip › biology-4636579-supplementary.pdf]

Multi-view Hypergraph Contrastive Learning for Uncertainty-Aware circRNA-Disease Association Prediction

Yu-Hao Zhou, Xiao-Peng Ji, Zhu-Hong You, Lei Wang, Chang-Qing Yu

Supplementary Materials

Table S1. Repeated negative-sampling evaluation.

Evaluation setting	AUC	AUPR
Original negative sample set	0.9687	0.9623
Ten random negative-sampling repetitions	0.9552 ± 0.0042	0.9301 ± 0.0099

Using the original five-fold models with positive samples fixed, ten random negative-sampling evaluations were conducted at a positive:negative ratio of 1:1. The original setting reports the five-fold mean. For repeated sampling, results are the mean ± population SD of the ten evaluation-specific five-fold means.

Table S2. Class-ratio reweighting analysis.

Total positive:negative evaluation weight	AUPR with gated fusion (without calibration)	AUPR with uncertainty-aware calibration
1:1	0.9111 ± 0.0328	0.9623 ± 0.0174
1:2	0.8422 ± 0.0515	0.9314 ± 0.0316
1:3	0.7855 ± 0.0639	0.9050 ± 0.0437
1:5	0.6967 ± 0.0784	0.8613 ± 0.0635
1:10	0.5533 ± 0.0905	0.7831 ± 0.0979
1:20	0.4064 ± 0.0900	0.6880 ± 0.1368
1:50	0.2509 ± 0.0786	0.5591 ± 0.1814

AUPR was calculated from the original five-fold predictions using the total positive:negative evaluation-weight ratios listed in the table. Results are five-fold means ± population SD.

Table S3. Cold-start prediction results for circRNAs and diseases.

Cold-start entity	Output	AUC	AUPR
circRNA	MHM branch	0.8123 ± 0.1156	0.8757 ± 0.0796
circRNA	HHN branch	0.7977 ± 0.0980	0.8531 ± 0.0669
circRNA	Gated and final	0.8081 ± 0.0840	0.8723 ± 0.0528
Disease	MHM branch	0.7398 ± 0.0623	0.8130 ± 0.0334
Disease	HHN branch	0.6356 ± 0.2537	0.6960 ± 0.1949
Disease	Gated and final	0.7380 ± 0.0691	0.8125 ± 0.0356

Leave-one-entity-out cold-start evaluation was conducted separately for circRNAs and diseases, with 54 and 300 test pairs, respectively, at a positive:negative ratio of 1:1. Association-derived inputs were constructed from positive training associations, and models were selected using independent validation sets. Results are means ± population SD across the evaluated entities. The gated-fusion and final-output results are identical at the reported precision and are therefore shown together.
